# Flexible Electrohydrodynamic Fluid-Driven Valveless Water Pump via Immiscible Interface

**DOI:** 10.34133/cbsystems.0091

**Published:** 2024-02-05

**Authors:** Zebing Mao, Naoki Hosoya, Shingo Maeda

**Affiliations:** ^1^ Department of Mechanical engineering, Tokyo Institute of Technology, Tokyo, Japan.; ^2^ Department of Engineering Science and Mechanics, Shibaura Institute of Technology, 3-7-5 Toyosu, Koto-ku, Tokyo 135-8548, Japan.; ^3^ Living Systems Materialogy (LiSM) Research Group, International Research Frontiers Initiative (IRFI), Tokyo Institute of Technology, Tokyo, Japan.

## Abstract

The conventional electrohydrodynamic (EHD) pump is limited to pumping functional and dielectric liquids, which restricts its applications in fields like microfluidics, food safety, and materials production. In this study, we present a flexible water pump driven by EHD fluid, achieved by integrating valveless elements into the fluidic channel. Our approach leverages the water–EHD interface to propel the immiscible aqueous liquid and reciprocate this process using the nozzle–diffuser system. All components of the water pump are digitally fabricated and assembled. The valveless parts are created using a laser cutting machine. Additionally, we develop a model for the EHD pump and nozzle–diffuser system to predict the generated flow rate, considering factors such as the asymmetrical performance of the EHD pump, pulse frequency, applied voltage, and structural parameters. Finally, we experimentally characterize the flow rates of both the EHD pump and water pump and apply the newly developed device to air bubble manipulation and droplet generation. This research broadens the range of specialized liquids pumped by EHD pumps to include other aqueous liquids or mixtures.

## Introduction

**Fig. 1.**
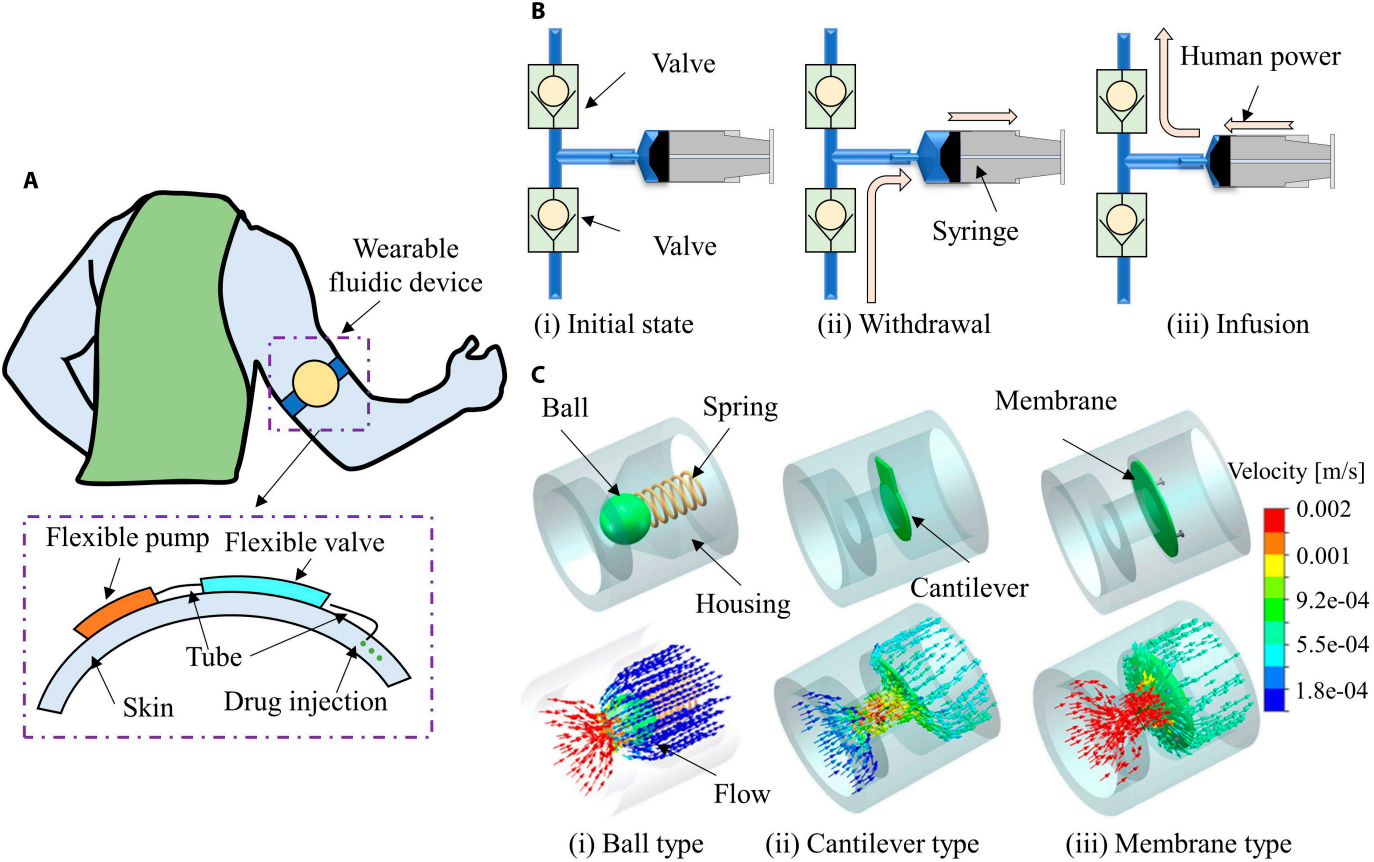
General conception diagram. (A) Wearable fluidic device. (B) Process of human-power-driven syringe integrated with 2 valves. (C) Three types of typical passive valves: ball type, cantilever type, and membrane type. (i) Ball type. (ii) Cantilever type. (iii) Membrane type.

Microfluidics has already made substantial contributions in various fields, such as diagnostics, food safety, and materials production [1–3]. Advancements in the field of biochemical sensor innovation, the crafting of devices, and the integration of technology, alongside the progress in low-power devices, have cleared the way for the actualization of wearable fluidic systems designed for the skin’s surface [4,5]. These systems possess the capability to examine biofluids that can be obtained from the epidermal layer, such as sweat and interstitial fluid (Fig. 1A). Pumps, as a crucial power source pushing fluids to the outer, have drawn increasing attention due to the need for miniaturization and integration in fluidic systems [6–8]. At present, microfluidic devices frequently employ commercial syringe pumps for fluid delivery. Nevertheless, these cumbersome syringe pumps exhibit specific constraints: (a) They possess a considerable dead volume owing to the dimensions of the syringe bore and connecting tubes. (b) Placing the entire system inside an incubator poses challenges. The complexity of fabricating and assembling conventional bulky syringe pumps, along with the presence of mechanical sliding parts, hinders their miniaturization. Consequently, there is a demand for developing on-board power sources that can offer high output power density without relying on mechanical sliding components. Small-size pumps, especially those capable of handling various fluids, hold great promise in addressing the mentioned challenges while satisfying the growing demands of microfluidics, lab-on-a-chip, and sample manipulation at small scales. Several small pumps, utilizing mechanisms such as pneumatic [9], chemical [10], electrostatic [11], piezoelectric [12], and electromagnetic [13] actuation, have been proposed. Similar to the human-power-driven syringe (Fig. 1B), these pumps work by contracting and expanding a flexible chamber, facilitating the movement of fluid.

To achieve this pumping process, 2 passive valves are essential. One valve holds the liquid in the chamber, while the other creates an orifice to control the liquid flow. During this process, the chamber undergoes repetitive volume changes, cyclically supplying the fluid, which results in periodic variations in flow rate and leads to a pulsating flow. The passive valves can be divided into 3 types: ball [14], cantilever [15], and membrane [16] (Fig. 1C). The cantilever valve consists of a flexible membrane or flap attached on one side, resembling a diving board or cantilever. The other end is free to move, and it acts as a movable lid or shutter [17]. When pressure is applied to one side of the valve, the flexible membrane bends or deflects, allowing fluid to flow through the valve. When the pressure is released, the membrane returns to its original position, closing the valve. The ball-type valve is a valve variant utilizing a spherical or ball-shaped closing element to regulate the fluid flow within a pipeline or system. The ball inside the valve has a hole (bore) through its center, which allows the fluid to pass when the hole is aligned with the valve’s inlet and outlet ports. Recently, some researchers have ingeniously designed tubes as soft kink valves, which provides a novel method to realize the controlling process of fluids [18,19]. However, achieving flexibility and easy fabrication in these valves is challenging due to the presence of moving components and the need for precision and tight tolerances in the design of the ball and its seating surfaces. Herein, we concentrate on utilizing an EHD (electrohydrodynamic) fluid and a valveless fluid rectifier to develop portable and flexible pumps. The utilized EHD fluid is generally a dielectric and operational liquid capable of producing a strong flow under the influence of a direct current voltage applied between positive and negative electrodes [20–23]. The EHD pump operates without the inclusion of any mechanical moving parts, ensuring a noise-free pumping process. This is accomplished through its dynamic pumping mechanism, wherein electric energy is directly transformed into kinetic energy, eliminating the necessity for mechanical components. EHD pumps have been applied in various domains such as serving as energy sources for propelling soft actuators/robots, facilitating microfluidics, and enhancing cooling systems over the recent decades [24–27]. Moreover, nozzle–diffuser systems are also commonly employed in pump designs [13,28]. A nozzle is a constricted passage that accelerates the flow of a fluid, typically a gas or liquid. Its main purpose is to enhance fluid velocity by decreasing the cross-sectional area of the flow path. A diffuser is the opposite of a nozzle. Therefore, we propose to develop a novel water pump by integrating the EHD pump with the nozzle–diffuser system.

## Mechanism, Design, and Fabrication

The EHD pump alone is not capable of directly driving the aqueous phase fluid. To address this limitation, we propose a method of supplying the aqueous solution by exploiting the water–EHD interface. In this approach, the fluid is not propelled by a mechanical piston but rather by the reciprocating action of the EHD pumps, which act as the power source. In our previous study, we have shown the feasibility of noncontinuous water pumping by harnessing the interface between the EHD fluid and the aqueous phase fluid [29].

### Mechanism

**Fig. 2.**
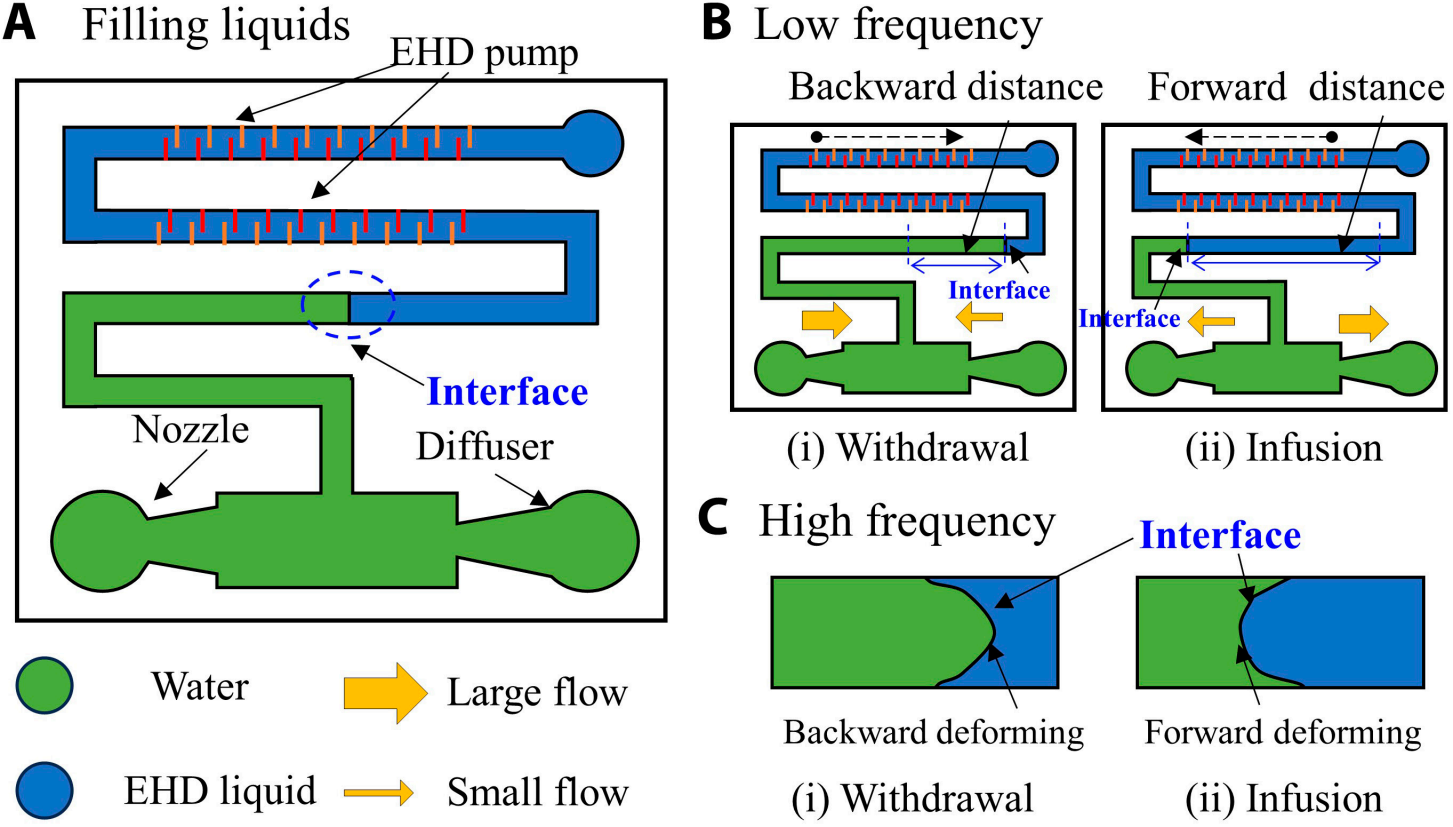
Principles of our proposed water pump. (A) Basic component of our valveless water pump. (B) Pumping mechanism of our water pump supplied by the applied voltage with low frequency. (C) Interface locomotion when the applied voltage with high frequency is provided.

The EHD fluid used in our system is an oil-type fluid that exhibits immiscibility with water, enabling the formation of an interface within the fluidic channel. This interface can be controlled and adjusted by moving it forward and backward using a bidirectional EHD pump. By leveraging this unique property, we can precisely regulate the flow of water and achieve efficient fluid manipulation without the need for direct contact with mechanical components. In our design, we have incorporated both a nozzle and a diffuser, which work together to facilitate fluid regulation (Fig. 2A). The water pump consists of a bidirectional EHD pump capable of propelling the EHD fluid in response to applied voltages, along with a diffuser, a nozzle, and inlet and outlet components. Once the aqueous solution and EHD fluid are filled, we proceed to apply a dc voltage to the EHD pump, generating a backward flow. Because of the interface, the water phase is also drawn along the fluidic channel. Considering the applied voltage with low frequency (Fig. 2B), the interface will move along the fluidic channel since the EHD pump can generate a certain amount of flow to push the interface. In the withdrawal process, the diffuser facilitates more forward flow, while the nozzle counteracts the backward flow, enabling the pump to draw water from the inlet. Subsequently, we reverse the direction of applied voltages, causing the EHD pump to generate forward EHD flow. Simultaneously, the water stream is moved to generate a short forward distance. During this stage, the diffuser prevents the water from returning to the inlet, while the nozzle assists in pumping the water toward the outlet. When the frequency becomes relatively high, the interface cannot move along the fluidic channel due to the limited flow generated by the EHD pump (Fig. 2C). At this time, the interface will deform with the direction of jet flow. In this case, the EHD pump works similarly to piezoelectric valveless pump pumps, which have a natural frequency to generate a maximum flow rate [29]. Moreover, in low-frequency situations, considering that our system operates at a relatively moderate speed, with a maximum velocity of approximately 5 mm/s, it is challenging for the EHD pump to disturb the movement of the interface during pumping process. In high-frequency scenarios, where the interface is achieved through vibration, there is no occurrence of disruption. If we were to use other liquids, their surface tension would differ. In our previous research [30], we found that surfactants (Span 80) are necessary to alter the liquid properties and the contact angle between the 2-phase liquids, thereby stabilizing the interface. Therefore, the water pump will decrease to nearly zero when the frequency becomes several times more than the natural frequency, in which the EHD pump cannot generate flow effectively and cannot deform or push the interface.

### Electrode spacing and fluidic channel width

The dimensions of the stacked pumps in our design were carefully considered based on the computational simulations. The criteria for selecting these dimensions were centered around maximizing flow rate. Since the overlapping distance of the electrode is 8 mm, we decided the fluidic channel width to be 8 mm. The fluid channel width was chosen to facilitate the maximum fluid flow rates, ensuring effective separation while avoiding excessive pressure drops.

**Fig. 3.**
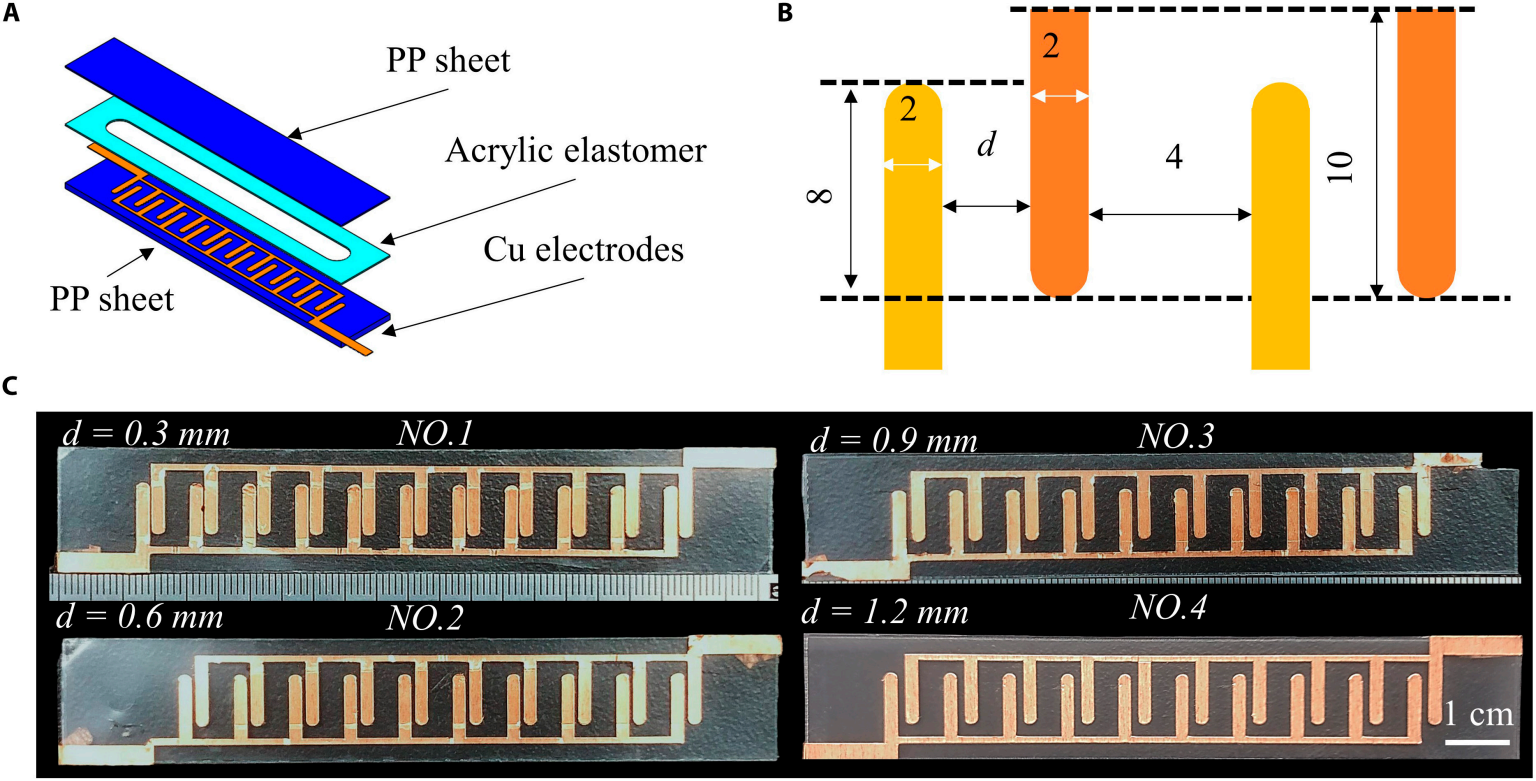
EHD pumps with 4 types of electrode gaps (*d*). (A) Sandwiched structures of the EHD pumps. (B) Layer out of the electrodes. (C) Four devices with different gaps (*d*).

**Fig. 4.**
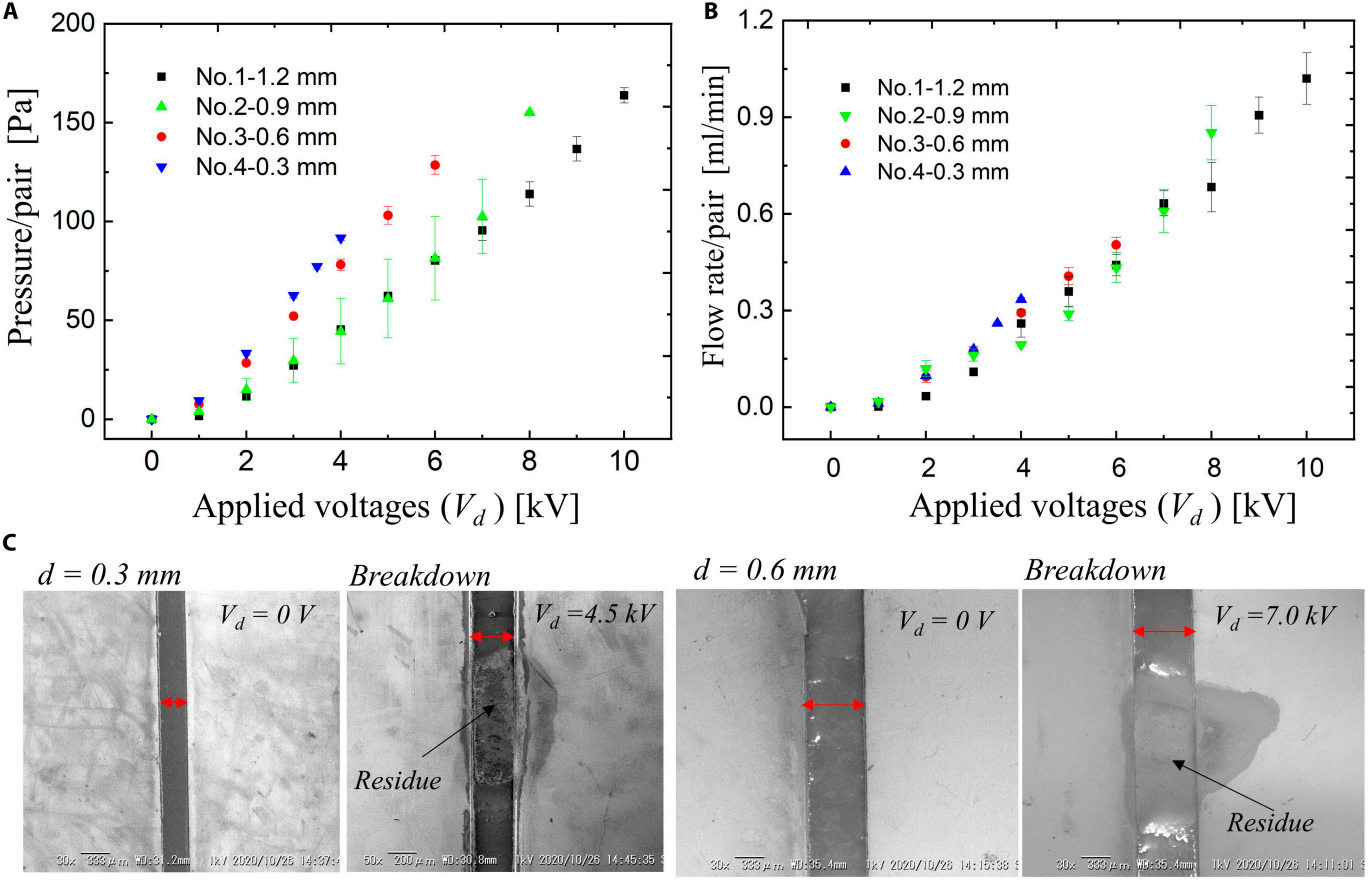
Performance of EHD pumps in terms of various electrode gaps. (A) Characteristics of pressure/pair versus applied voltages. (B) Characteristics of flow rate/pair versus applied voltages. (C) Breakdown occurs when the applied voltages are 4.5 kV (*d* = 0.3 mm) and 7 kV (*d* = 0.6 mm), respectively.

We checked the electrode spacing from the aspects of experimental results. We have prepared a device, consisting of 3 layers: polypropylene (PP) sheet, acrylic elastomer, and 10 pairs of Cu electrodes (Fig. 3A). The structure and dimensions of its electrodes are composed as illustrated in Fig. 3B. We have optimized the electrode gap (*d*) to facilitate the search for suitable pressure and flow output. We prepared 4 types of electrode gaps: 1.2, 0.9, 0.6, and 0.3 mm (Fig. 3C). Here, we made the distance between the electrode pairs to the constant value: 4 mm. The results are shown in Fig. 4. In Fig. 4A, the pressure varies as the electrode gap changes. The pressure increases as the electrode gap decreases. Taking the applied voltage of 4 kV for example, the generated pressure generated by the device with an electrode gap of 0.3, 0.6, 0.9, and 1.2 mm is 91, 78, 44, and 45 Pa, respectively. This might be caused by a small gap that is beneficial for the ionization process during the pumping process due to the short distance between 2 opposite electrodes. Additionally, we found that the gap has little influence on the generation of flow rate (Fig. 4B). This is due to the trade-off between the backflow between 2 pairs of electrodes and the increment of internal frictional losses. Moreover, the gap exerts effects on the working area (applicable voltages) of the EHD pumps. The No.1 (*d* = 0.6 mm) and No.2 (*d* = 0.3 mm) devices occur electric discharge when the applied voltages are 4.5 and 7 kV (Fig. 4C), respectively. After the electric breakdown, the electric gap is filled with conductive byproducts, which disabled the EHD pumps. Therefore, the small gap is detrimental to the working area of the EHD pumps. In this case, we choose the distance of 1.2 mm for our future research.

**Fig. 5.**
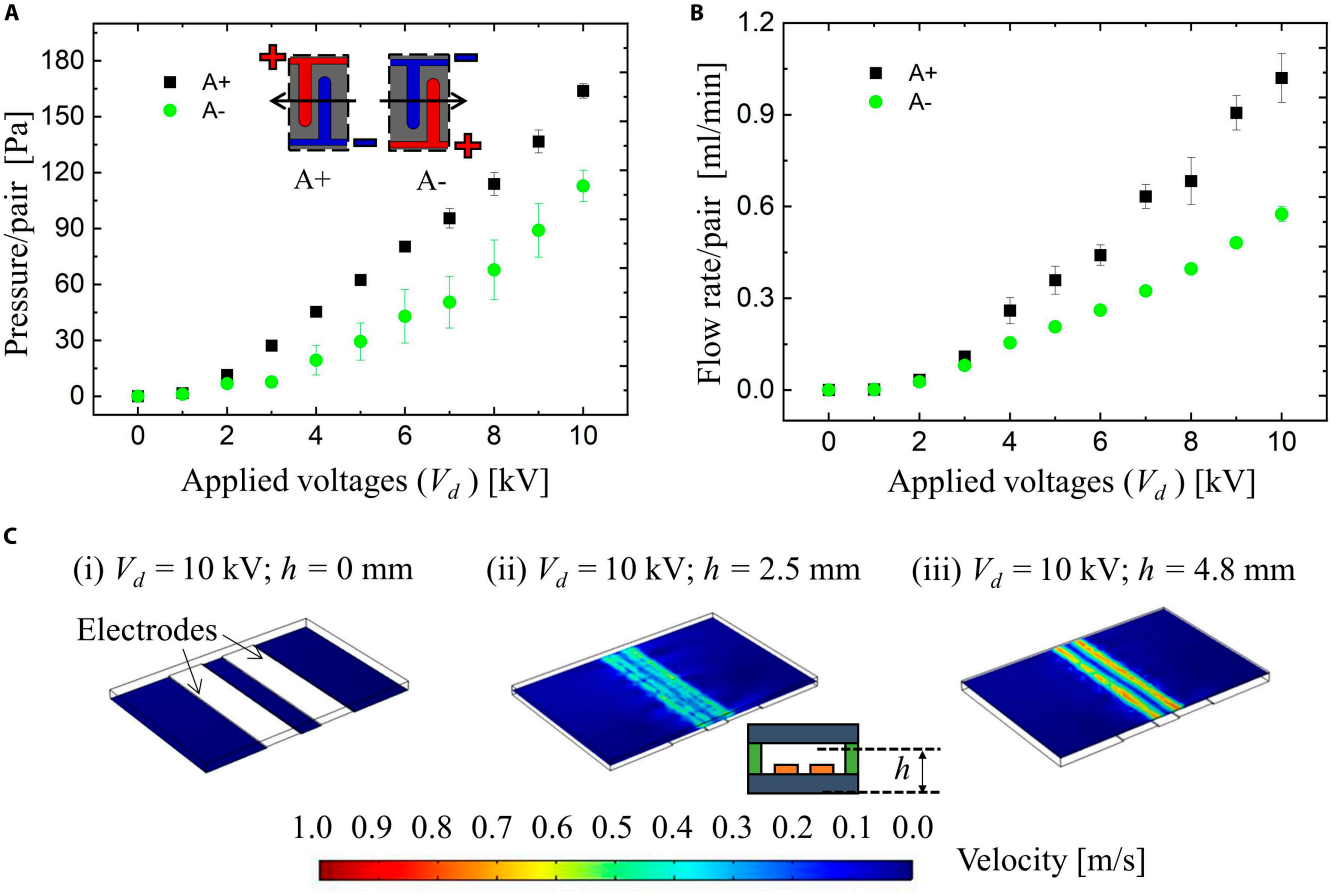
Experimental and simulation results of EHD pump. (A) Experimental results of EHD pumps in terms of pressure/pair. (B) Simulation results of EHD pumps in terms of flow rate/pair. (C) Flow field of EHD pumps with various channel heights.

Also, we conducted the simulation process and tested the pressure-flow rate characteristics in terms of pressure/pair and flow rate/pair (Fig. 5A and B). The fluidic flow of EHD pumps under an applied dc voltage is governed by the electric force ($\vec{F}$), which can be given by:

$$\vec{F}=q\vec{E}-\frac{\varepsilon_{0}}{2}E^{2}\nabla \varepsilon_{r}+\frac{\varepsilon_{0}}{2}\nabla \left(E^{2}\frac{\partial \varepsilon_{r}}{\partial \rho }\rho \right)$$ (1)

where *q*, *ρ*, $\vec{E}$, *ε*_0_, and *ε_r_* are charge density, the density of hydrofluoroether (HFE) 7300, electric field, the permittivity of vacuum, and the relative permittivity of HFE 7300, respectively. The electric forces include 3 types of forces: Coulomb force, dielectric force, and electrostriction force, which contributes to the pressure and flow rate of EHD pumps. In addition, the electric forces include 3 types of forces Coulomb force, dielectric force, and electrostriction force, which contributes to the pressure and flow rate of EHD pumps. e EHD flow can be described by the Navier–Stokes equation:

$$\frac{\partial \vec{\mu }}{\partial t}=-\left(\vec{\mu }\cdot \nabla \right)\vec{\mu }-\frac{1}{\rho }\nabla p+\frac{\eta }{\rho }\nabla^{2}\vec{\mu }+\frac{1}{\rho }q\vec{E}$$ (2)

where $\vec{\mu }$, *p*, and *η* are the velocity, pressure, and viscosity, respectively. According to Eq. 2, the flow of HFE 7300 is determined by the electric field and charges. The density, dielectric constant, and viscosity of HFE 7300 are 1,660 kg/m^3^, 6.1 and 1.18 mPa·s, respectively. We used the commercial software, COMSOL Multiphysics 5.6 (COMSOL Inc.), to numerically analyze the flow field. The 2 used modules were ac/dc module and the laminar flow module, respectively. Here, we only simulated one pair of copper electrodes and afterward obtained the generated pressure of EHD pumps. The simulation process was conducted by: (a) simulating the electric field of the electrodes, (b) calculating the volume force using the Eq. 1, and (c) simulating the generated pressure using the laminar flow based on the Eq. 2. For the boundary condition, we applied the dc voltages on one electrode from 0 to 10 kV, and the voltage of the other electrode was set to 0 kV. The pressure at the inlet and flow velocity at the outlet of the EHD pump were set to 0 Pa and 0 m/s, respectively (Fig. 5C).

### Design and fabrication

**Fig. 6.**
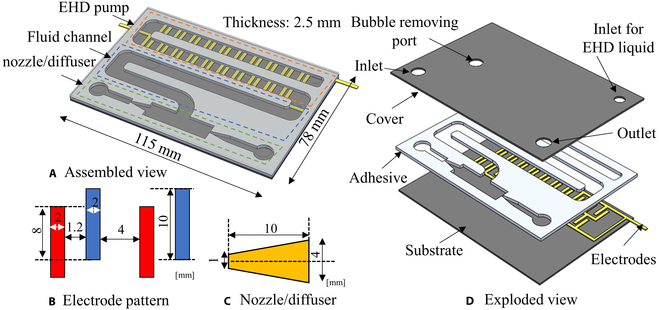
Design and dimensions of our device. (A) Assembled view of 3D modeling of our valveless water pump. (B) Electrode pattern of EHD pump. (C) Dimensions of nozzle and diffuser. (D) Exploded view of 3-layer sandwiched water pump.

**Fig. 7.**
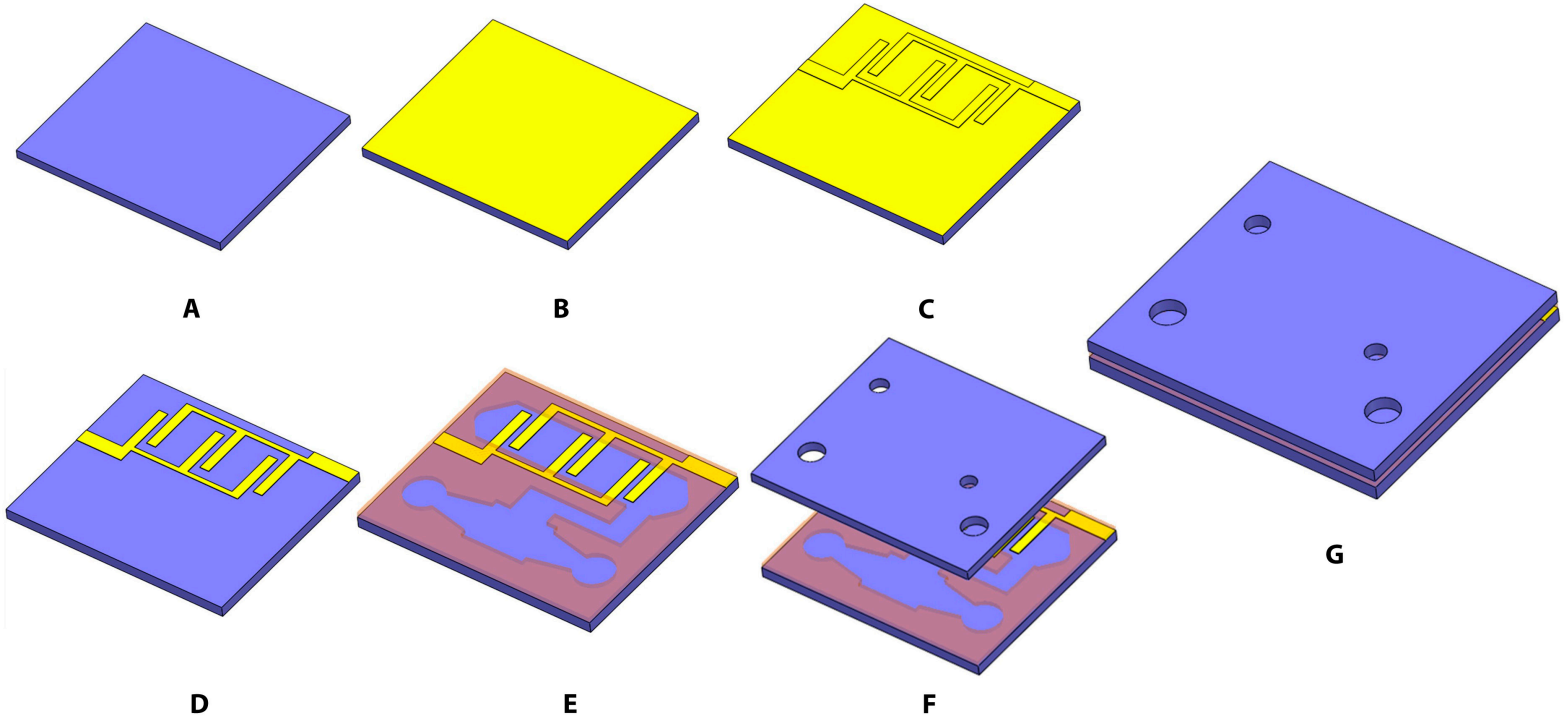
Fabrication process of the water pump. (A) Preparation and clean of PP sheet. (B) Attachment of the copper layer. (C) Patterning process of electrode layers. (D) Removal of unnecessary parts. (E) Laser cutting process of the fluidic channels. (F) Aligning and assembling process. (G) Final assembled device.

In our study, we utilized hydrofluoroethers (HFE 7300), a type of 3M Novec engineered fluid, and water dyed with red coloring as the pumping and aqueous liquids. The choice of HFEs was made because of their exceptional thermal and chemical stability. The planar valveless pump has 3 basic parts: an EHD pump with designed electrodes, a fluidic channel for a low-frequency pumping process, and a nozzle/diffuser part (Fig. 6A). The stacked pumps have overall dimensions of 115 mm in length, 78 mm in width, and 2.5 mm in height. The EHD pump is designed with 20 pairs of parallel plate electrodes. By using the serialized electrode patterns, we can increase the pressure. On the other hand, we designed the width of the fluidic channel to be 8 mm to ensure an adequate flow rate. The electrode is designed with an overlapping length of 8 mm and an electrode gap of 1.2 mm. The fluidic channel has a height of 0.5 mm. The separation between 2 pairs of electrodes is configured at 4 mm (Fig. 6B). The fluidic channel in the middle section, functioning as the reservoir for establishing the interface between the EHD fluid and water, is designed to be approximately 150 mm. In our design, the nozzle/diffuser features a throat width of 1 mm, a length of 10 mm, and an initial width of 4 mm (Fig. 6C). The flexible water pump comprises 3 layers: substrate, fluidic channel, and cover (Fig. 6D). Both the base and cover components were constructed using PP sheets, while the electrodes were crafted from copper. The fluidic channel was formed using a layer of acrylic elastomer (3 M VHB4910J) with a thickness of 0.5 mm. To address the issue of bubbles during the liquid filling process, which can significantly impede fluid flow and efficiency, causing undesirable fluctuations and reducing overall pump functionality, a new port was designed for bubble removal. For connecting with the outer tank, 2 adaptors were produced using a 3D printer (Agilista-3200, Keyence) and composed of transparent resin (AR-M2, Keyence). The fabrication process involved the following steps (Fig. 7): (a) A4-sized PP sheets were cleaned using acetone and deionized water. (b) A layer of copper (Cu) was adhered to the PP sheets. (c) The seed layer of electrodes was formed using a cutting plotter (GRAPHTEC CE6000-40 Plus). (d) Unnecessary parts of the copper sheet were removed to obtain the electrode pattern. (e) The copper sheet was then divided into individual substrates for the EHD pumps using a laser cutter. (f) The fluidic channel was prepared using the laser cutter and attached to the substrate of the EHD pumps. (g) The cover was fabricated and aligned with the prepared substrate.

## Modeling, Experiments, and Results

In this chapter, our emphasis is on modeling the produced liquid volume of the water pump and characterizing the performance of both the EHD pump and the water pump. We delve into the analysis of flow rates achieved under varying applied voltages and frequencies on the planar water pump.

### Characterization of EHD pump

**Fig. 8.**
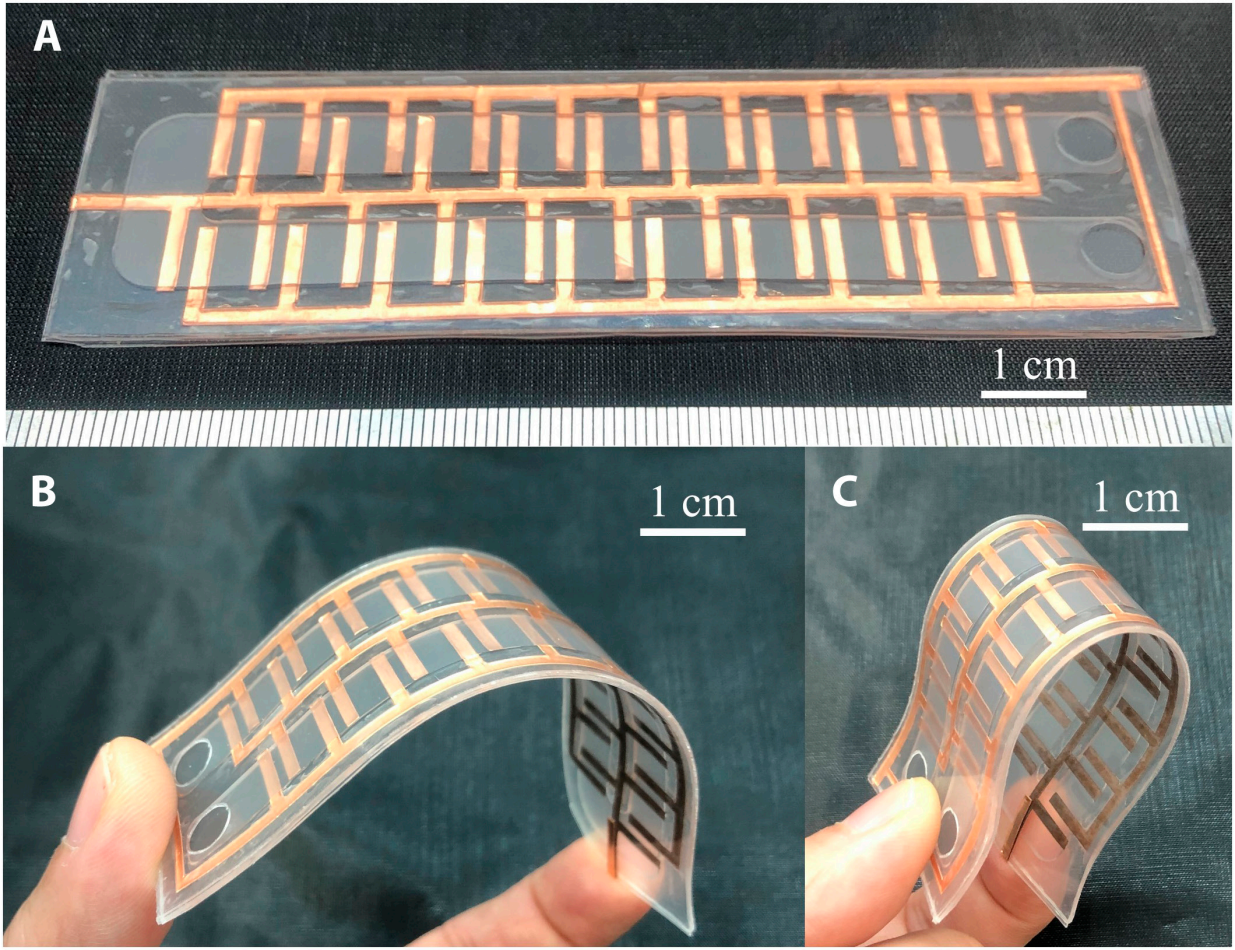
Fabricated EHD pump. (A) Photos of EHD pump. (B) Bending state of EHD pump. (C) Squeezed state of EHD pump.

**Fig. 9.**
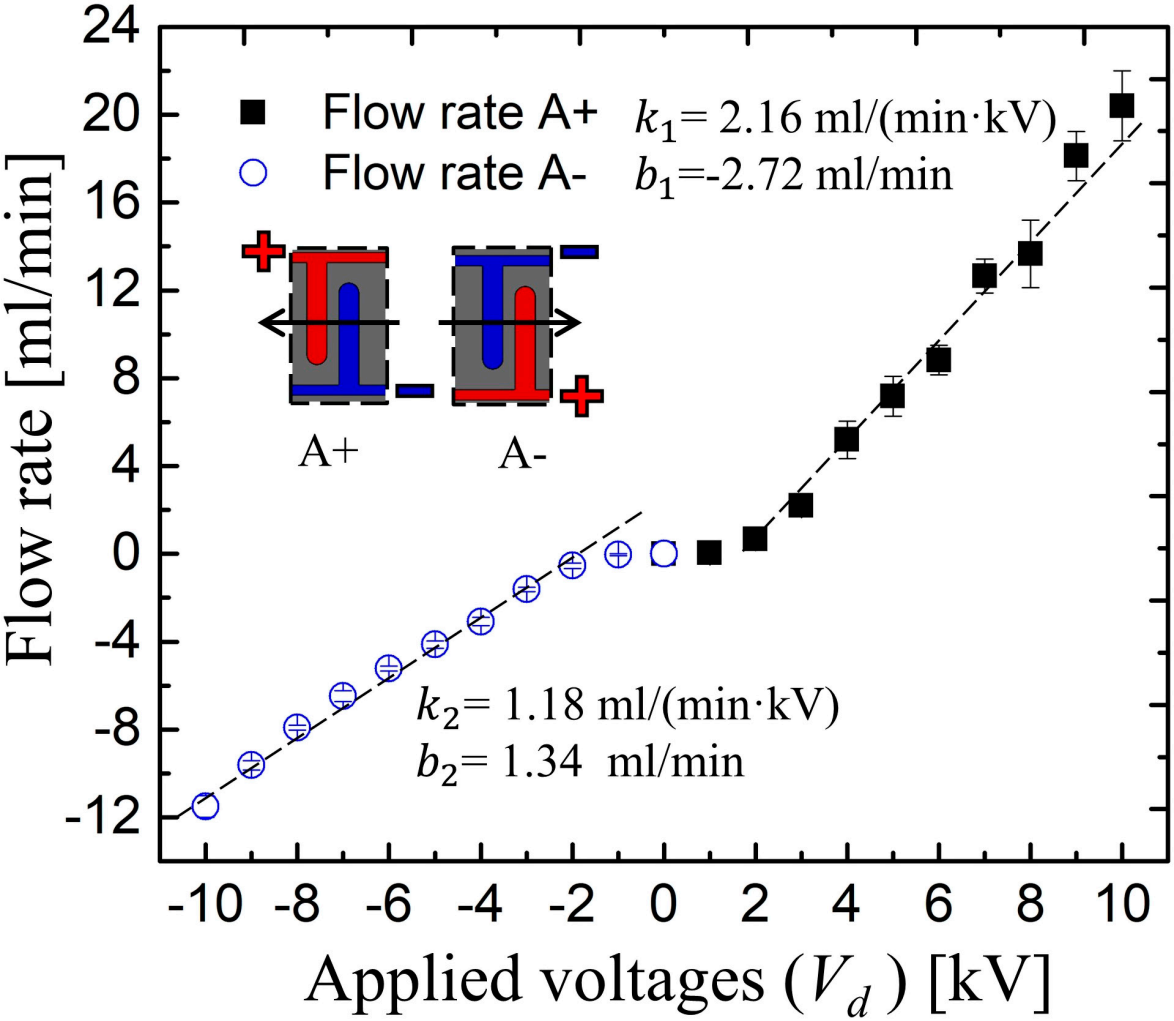
Flow rate characterization of EHD pump varies with the growth of applied voltages. A+ and A− indicates the infusing and withdrawing direction in our water pump, respectively.

Figure 8 depicts the bendable and squeezable EHD pump. To enhance the flow rate of the EHD pump, we incorporated 2 additional columns of electrodes. Upon fabrication, the EHD pump exhibited bending and squeezing behaviors, facilitated by the use of soft and flexible materials and thin copper layers in our study. The flow rate characteristics of this pump were examined, as depicted in Fig. 9. In this investigation, the applied voltage varied from 0 to 10 kV in 2 directions. We designated the EHD flow from the right side to the left side as positive flow (labeled as A+), and conversely, as negative flow (labeled as A−). Both flow rates demonstrated an increase as the applied voltages increased, aligning with findings presented in our earlier publication [31]. The maximum generated flow rates in our pumps were 20.39 ml/min (A+, SD [standard deviation]: 1.59) and −11.51 ml/min (A−, SD = 0.50) at 10 kV, respectively. Additionally, the difference in flow rates between the 2 directions fluctuated with the applied voltages, reaching around 43.55% at 10 kV. For the EHD pump, we apply square wave voltage whose amplitude is *V*_0_ and frequency is *f*:

Vt=V0,nT<t≤nT+T/2−V0,nT+T/2<t≤n+1T (3)

where *t* is time, *T* = 1/*f* is period, and *n* is integer. Voltage dependence of generated pressure by EHD pump, it even exhibits asymmetrical performance when reversing sign of voltage despite symmetrical structure of electrodes [32]. Here, we phenomenologically assume linear relation as:

$$Q_{+}=k_{1}V_{0}+b_{1}\left(Q_{+}>0\right)$$ (4)

$$Q_{-}=-k_{2}V_{0}+b_{2}\;Q_{-}<0$$ (5)

where *Q*_+_, *Q*_−_, *V*_0_ are forward flow rate, backward flow rate and applied voltages respectively. *k*_1_, *b*_1_, *k*_2_, and *b*_2_ are the slopes and intercepts for the linear relationship for the applied voltages and flow rates. According to the equations above, we could obtain the slopes and intercepts for the infusion and withdrawal process, which are *k*_1_= 2.16 ml/(min·kV), *b*_1_ = −2.72 ml/min, *k*_2_ = 1.18 ml/(min·kV), and *b*_2_ = 1.34 ml/min.

### Modeling for pumped volume

**Fig. 10.**
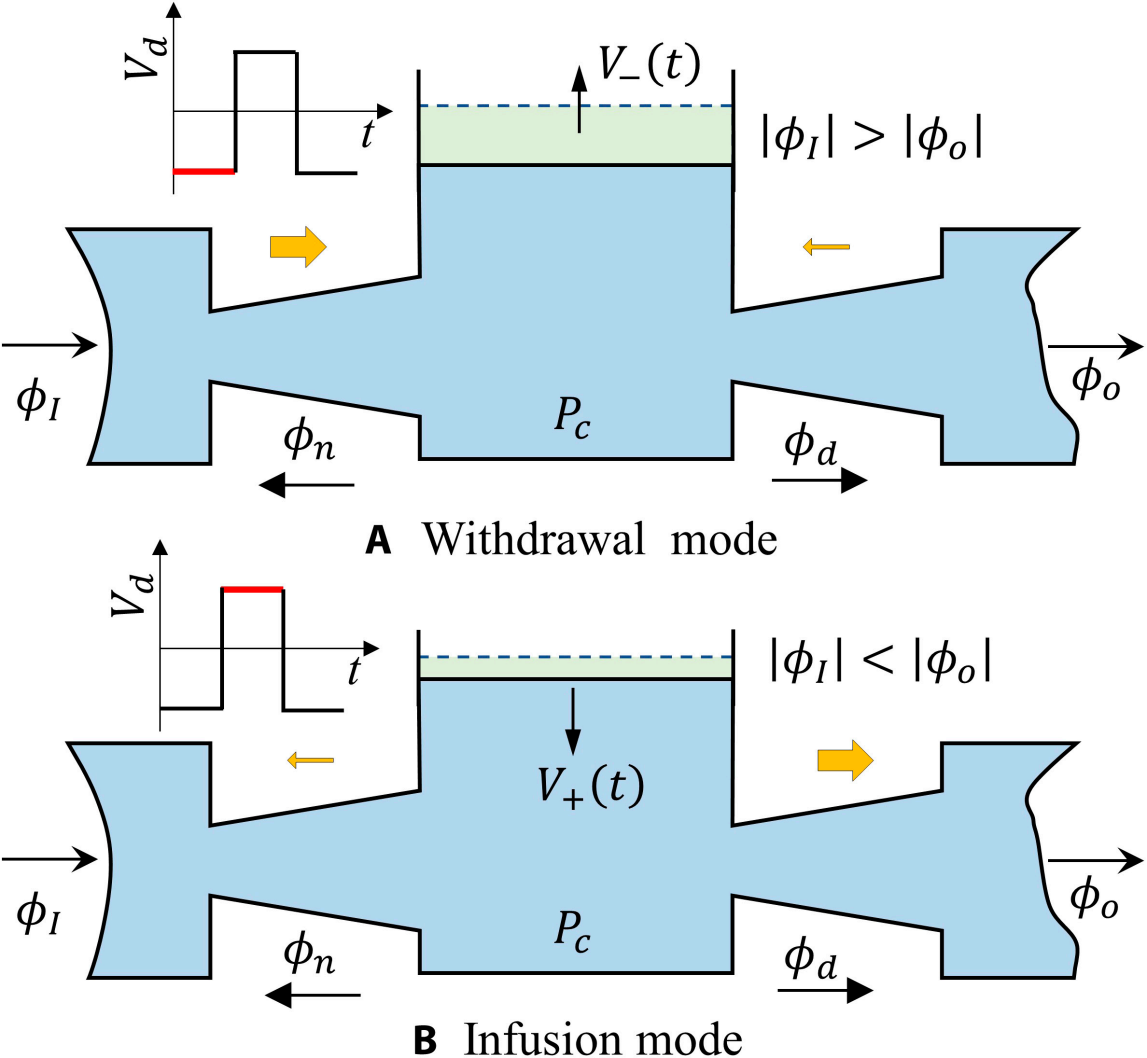
Schematics of modeling the water pump. (A) Withdrawal mode. (B) Infusion mode.

Here, we define the cross-sectional throat area of the diffuser and nozzle to *A* and the water density to *ρ* (Fig. 10). Assuming the pressure in the fluidic channel to be *P_c_*, we can obtain the volume flows in the diffuser and nozzle direction [33,34]:

$$\phi_{d}=C/{\left(\xi_{d}\right)}^{1/2}$$ (6)

$$\phi_{n}=C/{\left(\xi_{n}\right)}^{1/2}$$ (7)

where *C* = *A*(2*P_c_*/*ρ*)^1/2^, *ξ_d_* and *ξ_n_* are pressure-loss coefficients of the diffuser and the nozzle respectively. *ρ* is water density. Due to geometrical difference of the diffuser and nozzle, *ξ_d_* ≠ *ξ_n_* and it enables the device to generate asymmetrical flow. Considering the applied frequency, we could obtain the flow rate [35]:

$$Q_{+}\left(t\right)=Q_{+}\left(1-e^{-\frac{t}{T_{c}}}\right)\;\mathrm{nT}<t\leq \mathrm{nT}+T/2\;$$ (8)

$$Q_{-}\left(t\right)=Q_{-}\left(1-e^{-\frac{t}{T_{c}}}\right)\;\mathrm{nT}+T/2<t\leq \left(n+1\right)T\;$$ (9)

where *Q*_+_(*t*), *Q*_−_(*t*), *T_c_* are forward flow rate and backward flow rate varying with time and the time constant, respectively. Here, we have chosen a time constant value of 0.03, as it closely aligns with the reported literature [35], considering the permittivity, density, and thermal conductivity of the liquid used in this study.

#### Withdrawal mode

At this stage, the chamber volume is increasing, *Q* = *dV*(*t*)/*dt* > 0. This leads to a net flow into the chamber. The inlet element functions as a diffuser, while the outlet element serves as a nozzle. The flows at the inlet and outlet can be characterized, considering the volume flow into the diffuser through the inlet (*ϕ_I_*) and the volume flow out of the nozzle through the outlet (*ϕ_o_*). Then, we can derive the net volume flow according to the references:

$$\phi_{I}=\phi_{d}=C/{\left(\xi_{d}\right)}^{1/2}$$ (10)

$$\phi_{o}=-\phi_{n}=-C/{\left(\xi_{n}\right)}^{1/2}$$ (11)

$$\phi_{I}-\phi_{o}=\phi_{d}+\phi_{n}$$ (12)

A net chamber flow can be obtained:

$$\phi_{I}-\phi_{o}=\frac{d\left(V\left(t\right)\right)}{\mathrm{dt}}=-Q_{-}\left(t\right)$$ (13)

$$\phi_{d}+\phi_{n}=C\left({\left(\xi_{d}\right)}^{-1/2}+{\left(\xi_{n}\right)}^{-1/2}\right)$$ (14)

Then, the value of *C* can be derived:

$$C=Q_{-}\left(t\right)/\left({\left(\xi_{d}\right)}^{-1/2}+{\left(\xi_{n}\right)}^{-1/2}\right)$$ (15)

Therefore, the outlet flow can be rewritten:

$$\phi_{o-\text{withdrawal}}=-\phi_{n}=Q_{-}\left(t\right)/\left(1+\sqrt{\eta }\right)$$ (16)

where *η* = *ξ_n_*/*ξ_d_*. With the given dimensions of the nozzle/diffuser assembly and assuming an equal pressure difference during both positive and negative actuation, we can calculate the efficiency (*η*) of the nozzle/diffuser structure using the following equation [36]:

$$\eta =\frac{19}{20}{\left(\frac{W_{2}}{W_{1}}\right)}^{0.34}$$ (17)

where inlet width (*W*_1_) and outlet width (*W*_2_) of the diffuser are 1 and 4 mm in our study, respectively. Therefore, we can calculate the value of *η* is 1.30.

#### Infusion mode

At this stage, the chamber volume is decreasing, *Q* = *dV*(*t*)/*dt* < 0, leading to a net flow out of the chamber. The inlet element functions as a nozzle, while the outlet element serves as a diffuser. The flows at the inlet and outlet can be described:

$$\phi_{I}=-\phi_{n}=-C/{\left(\xi_{n}\right)}^{1/2}$$ (18)

$$\phi_{O}=\phi_{d}=C/{\left(\xi_{d}\right)}^{1/2}$$ (19)

$$C=Q_{+}\left(t\right)/\left({\left(\xi_{d}\right)}^{-1/2}+{\left(\xi_{n}\right)}^{-1/2}\right)$$ (20)

$$\phi_{O-\text{infusion}}=Q_{+}\left(t\right)\sqrt{\eta }/\left(1+\sqrt{\eta }\right)$$ (21)

Assuming the pressure-loss coefficients (*ξ_d_* & *ξ_n_*) to be constant throughout the pump cycle, the pumped volume during a period can be derived:

$$\begin{array}{c} V=\int_{0}^{T/2}\phi_{o-\text{withdrawal}}\mathrm{dt}+\int_{T/2}^{T}\phi_{O-\text{infusion}}\mathrm{dt}=\frac{T}{2}\frac{\left(Q_{-}+\sqrt{\eta }Q_{+}\right)}{1+\sqrt{\eta }}+\\ \frac{T_{c}}{\left(1+\sqrt{\eta }\right)}\left[Q_{-}\left(e^{-\frac{T}{2T_{c}}}-1\right)+\sqrt{\eta }Q_{+}\left(e^{-\frac{T}{T_{c}}}-e^{-\frac{T}{2T_{c}}}\right)\right] \end{array}$$ (22)

the generated flow rate can be written by substituting the Eqs. 4, 5, 8, and 9 into Eq. 22:

$$\begin{array}{c} Q=\frac{V}{T}=V_{0}\left[\frac{\sqrt{\eta }k_{1}-k_{2}}{2\left(1+\sqrt{\eta }\right)}-\frac{T_{c}fk_{2}}{\left(1+\sqrt{\eta }\right)}\left(e^{-\frac{1}{2{\text{fT}}_{c}}}-1\right)+\sqrt{\eta }k_{1}\left(e^{-\frac{1}{fT_{c}}}-e^{-\frac{1}{2{\text{fT}}_{c}}}\right)\right]+\\ \frac{b_{2}+b_{1}\sqrt{\eta }}{2\left(1+\sqrt{\eta }\right)}+\frac{T_{c}fb_{2}}{\left(1+\sqrt{\eta }\right)}\left(e^{-\frac{1}{2{\text{fT}}_{c}}}-1\right)+\frac{T_{c}fb_{2}}{\left(1+\sqrt{\eta }\right)}\sqrt{\eta }b_{1}\left(e^{-\frac{1}{fT_{c}}}-e^{-\frac{1}{2{\text{fT}}_{c}}}\right) \end{array}$$ (23)

where the applied frequency *f* = 1/*T*, in which *T* is the period of applied voltage signal. We can obtain the limit for the Eq. 23:

$$\underset{f\to 0}{\text{lim}}Q=\frac{\left(\sqrt{\eta }k_{1}-k_{2}\right)V_{0}+b_{2}+b_{1}\sqrt{\eta }}{2\left(1+\sqrt{\eta }\right)}$$ (24)

$$\underset{f\to \infty }{\text{lim}}Q=\frac{\left(\sqrt{\eta }k_{1}-k_{2}\right)V_{0}+b_{2}+b_{1}\sqrt{\eta }}{2\left(1+\sqrt{\eta }\right)}$$ (25)

### Characterization of water pump

**Fig. 11.**
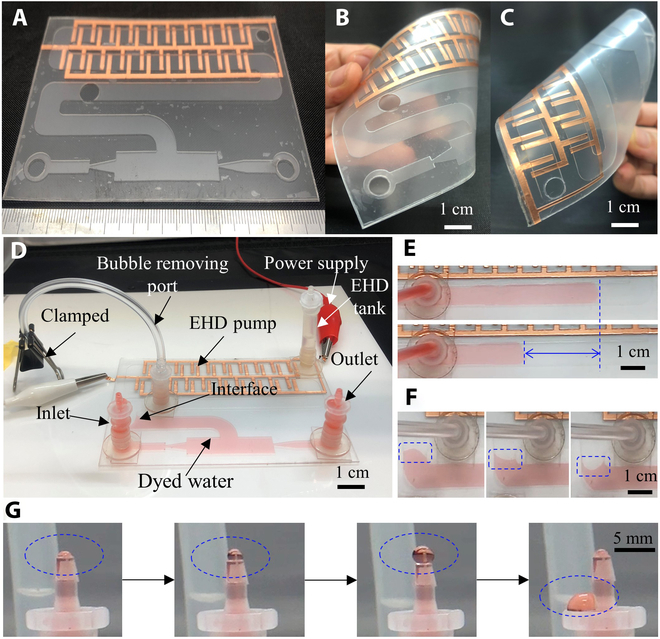
Experimental results of our valveless water pump. (A) Photo of the fabricated water pump. (B) Bent state. (C) Pinched water pump. (D) Experimental setup. (E) Locomotion of interface when the pump is powered under the voltage with low frequency. (F) Locomotion of interface at high frequency. (G) Push out a droplet to the outlet.

The fabricated water pump is planar and thin as shown in Fig. 11A. The water pump exhibits deformability in response to external forces although it has a relatively large area (Fig. 11B and C). In Fig. 11D, high-voltage amplifier is used to supply the power for the EHD pump to drive the transparent EHD fluid. To provide the reservoirs, we used 3 short tubes so that the pump can easily realize the portability and replacements. The bubble-removing ports were clamped when the filling process is finished. Here, we used the dyed water so that we could observe the interface easily. When the applied voltage is set to 4 kV and 0.1 Hz, we can observe the interface moves along the fluidic channel (Fig. 11E). This is because the EHD pump can generate a strong and powerful flow stream at a certain amount of time when the frequency is low. This power is transmitted to water via the interface and then pushes the water stream to move. As the applied voltage and frequency are switched to 4 kV and 10 Hz, the locomotion of the interface changes to the work pattern that is similar to the membrane vibration (Fig. 11F). At this stage, it can be observed that the interface cannot move in a long distance that occurred in (Fig. 11E) since the EHD pump cannot generate enough flow stream in a short period time. As a result, the interface deforms in a crescent shape. In Fig. 11G, the flexible planar water pump was successfully demonstrated to effectively pump an aqueous droplet to the exterior.

**Fig. 12.**
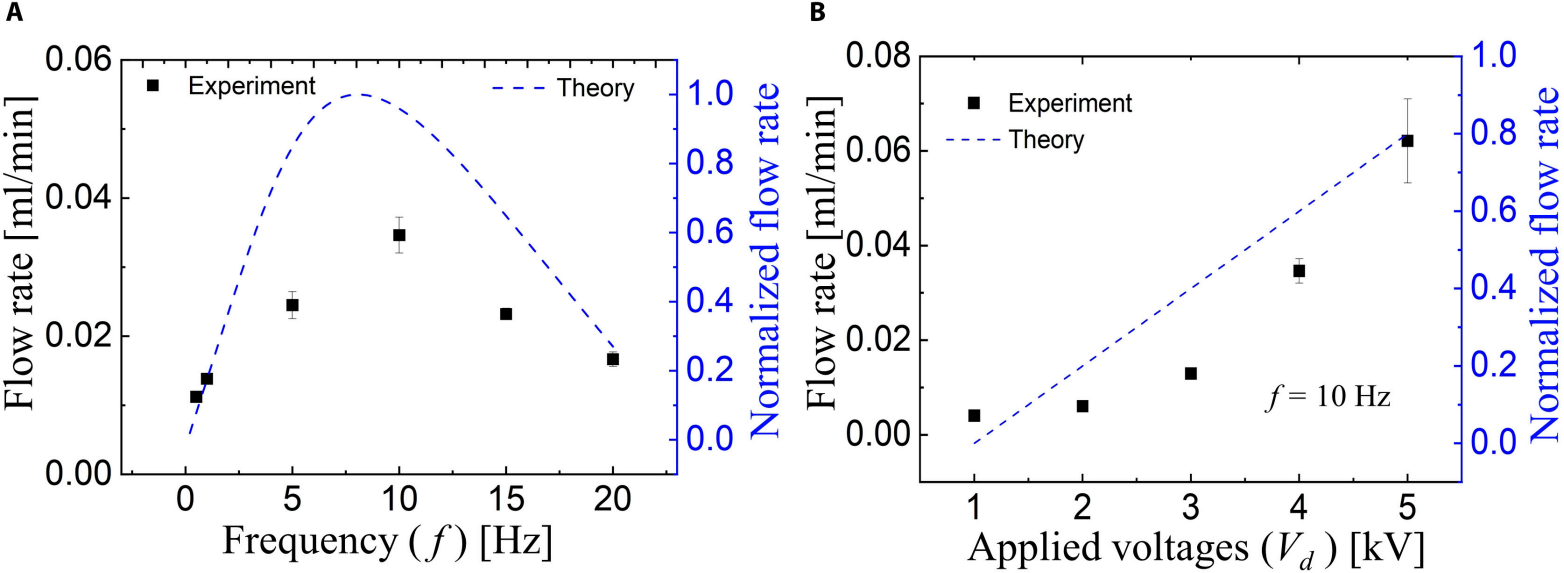
Flow rate characteristics of our valveless water pump. (A) Flow rate of the water pump under the applied voltage of 4 kV and 50% duty versus the frequency. (B) Flow rate of the water pump under the constant frequency of 10 Hz and 50% duty versus the applied voltages.

Figure 12 illustrates the volume flow rate of the valveless water pump under the conditions of an applied voltage and pulse duty of 4 kV and 50%, respectively (refer to Movies S1 and S2). The findings indicate that the flow rate initially increases up to a certain frequency (10 Hz) and subsequently starts to decrease. The maximum flow rate of 0.03 ml/min (SD = 0.002) is attained when the frequency is set at 10 Hz (Fig. 12A). In line with Eq. 23, it can be observed that the flow rate approaches 0 as the frequency tends toward both 0 and infinity. Therefore, the flow rate is expected to rise to the maximum value as the frequency increases and then decrease to the minimum value, representing the first term in Eq. 23.

The prediction following Eq. 23 agrees with the tendency that the experiments show. We wrote the Python code to estimate the value by using the obtained values of *η*, *k*, *k*_1_, *k*_2_, *b*_1_, and *b*_2_. The results show that the predicted curve agrees with the tendency that experimental results show. The maximum flow rates based on our theoretical model also happen at 8.1 Hz compared with the experimental one (10 Hz). The error might originate from the assumption that the ratio of coefficients of pressure loss of the nozzle and diffuser remains constant during operation. Variations in operating conditions and fluid dynamics could lead to fluctuations in this ratio, impacting the flow rate and frequency. Also, the amplitude difference between theoretical and experimental flow rate is because we neglected factors such as energy losses from pressure and velocity at the immiscible interface, as well as frictional resistance within the fluidic channel. Furthermore, we investigated the relationship between the applied voltages and flow rate, as shown in Fig. 12B. The results indicate a substantial increase in flow rate with increasing applied voltage. Theoretical values are calculated, and they roughly align with the experimental data. In the theoretical model, the flow rate is expected to increase linearly with the applied voltage while the experimental results show it follows a parabola-like pattern. This is probably because this model overlooks the influence of pressure during the infusion and withdrawal modes, which could play substantial roles and contribute to the observed deviations between theory and experiment. Another problem is that air bubbles probably cause both the errors in Fig. 12A and B because the oscillation of the interface can easily generate the tiny bubbles in the fluidic channels. Finally, according to our experimental result, the threshold frequency value to determine the transition interface from a plane to a membrane vibration is about 5 Hz.

## Applications

**Fig. 13.**
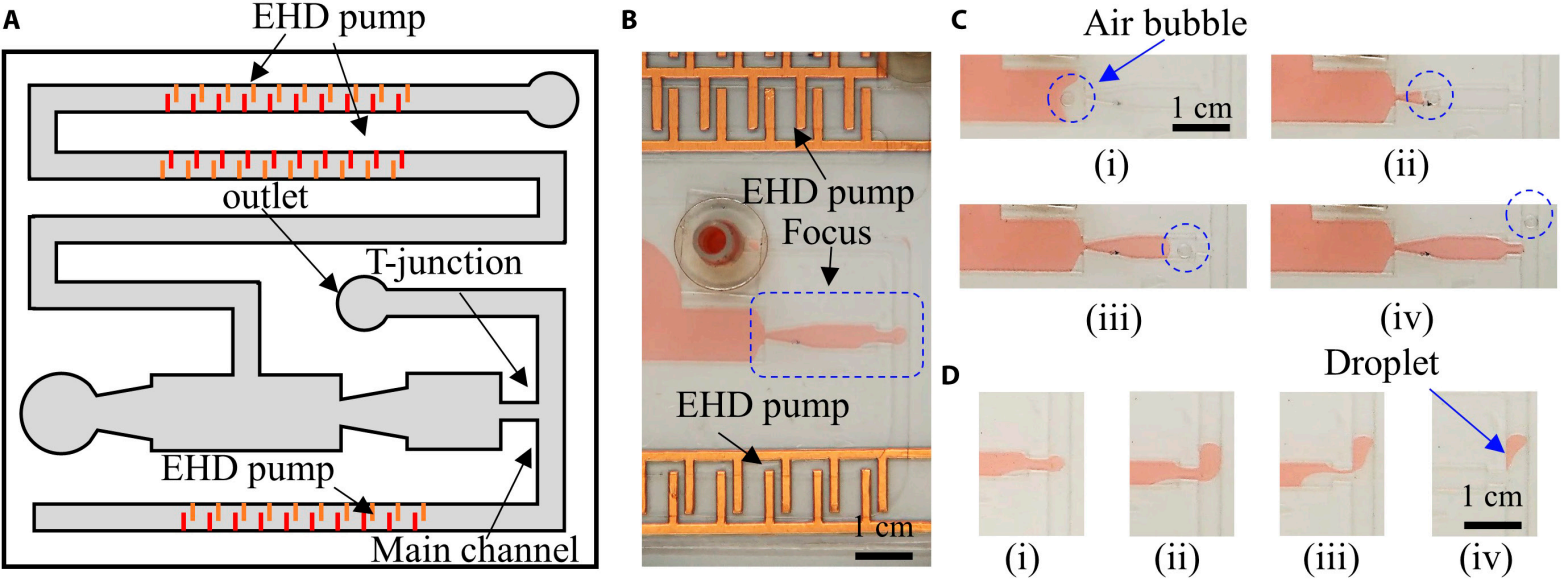
Applications of our valveless water pump. (A) Design for manipulation of air bubbles and droplet generation using our developed planar water pump. (B) Optical image of the devices. (C) Pushing an air bubble in the EHD fluid via the interface in the fluidic channel using the flow generated by the water pump. (D) Droplet generation based on the simple T-junction using another EHD pump and our developed water pump.

We used the valveless planar water pump to demonstrate the manipulation of air bubbles in the 3-phase interface and water in oil droplet generation using the T-junction structure (Fig. 13 and Movie S3). Figure 13A shows the design of the new devices. Another EHD pump is incorporated into the planar water pump since it is easy to be integrated easily. This pump is used to provide the oil phase fluid to one inlet of the T-junction droplet generator. The water phase fluid is supplied by the water pump in this study. The actual device is shown in Fig. 13B. We only focus on the T-junction structure. The applied voltage of the water pump is 4 kV with a 10-Hz frequency square wave. The device can be used to manipulate the air bubble, which is located at the interface between the dyed water and EHD fluid (Fig. 13C(i)). As the pumping process continues and the interface was moved, the air bubble is inserted into the channel of the water pump near the outlet (Fig. 13C(ii) and (iii)). After the air bubble was totally inserted into the main channel, we powered on the EHD pump in the bottom part and thus generating the EHD jet flow, which cause the air bubble to move the main fluidic channel (Fig. 13C(iv)).

Furthermore, we used the newly developed device to generate the water in oil droplets in the lab. We first pump the water to the main channel using the water pump (Fig. 13D(i)). After a stream of water plug is inserted into the main channel (Fig. 13D(ii)), we switch on the EHD pump at the bottom and thus causing a jet to cut the water plug (Fig. 13D(iii)). Finally, a water droplet is formed (Fig. 13D(iv)). In contrast with our previous device [30], our new device does not require a refilling process after the water tank was exhausted. Due to its planar and flexibility, it has a substantial protentional to be used in wearable fluidic devices.

## Conclusion

In this paper, we present the design, fabrication, and characterization of a flexible valveless water pump utilizing EHD principles. The proposed flexible water pump comprises PP sheets, a VHB (Very High Bond) fluidic channel, and Cu (Copper) electrodes, all of which contribute to its flexible properties. The overall dimensions of the water pump are 115 × 78 × 2.5 mm^3^. The measured pumping flow rates reach 20.39 and 11.51 ml/min in 2 directions when the pump is powered at 10 kV. Moreover, we have successfully established an interface between the EHD fluid and water, enabling the water pump to achieve a flow rate of 0.03 ml/min at an applied voltage of 4 kV and a frequency of 10 Hz. We also model this type of pump considering the applied voltage, frequency, and hydraulic dynamics of the nozzle/diffuser system and compare the experimental and simulation results, which show a high-level agreement. Furthermore, we apply the flexible, planar, and valveless water pump to manipulate the air bubble in the fluidic channel and generate water in oil droplets. To summarize, our work demonstrates the capability of the EHD pump to efficiently pump aqueous solutions through the integration of nozzle–diffuser flow regulation and an immiscible liquid interface. This flexible valveless water pump holds potential for various applications in microfluidics, lab-on-a-chip devices, and other fields requiring precise and controllable fluid pumping. The pump needs the supplied voltage on the order of kilovolts. The electrical current was produced by the pump and established that it remains below the threshold of 20 uA, which is negligible risk of harm to the human body. However, particularly in the face of unforeseen or extreme circumstances, it is customary to install insulation layers, such as the plastic and gauze, at the base of the device. In the future, we will utilize our pump to administer glucose infusion, guided by the feedback signal from an adhesive blood glucose sensor.

The EHD wearable fluidic system, while innovative, is not without its substantial shortcomings. One primary limitation lies in its dependency on external high-voltage power sources. The need for a continuous power supply, typically from high-voltage circuits, restricts the system's autonomy and portability, hindering its effectiveness in certain applications. Additionally, the complexity of the fluidic components and the associated control systems can contribute to increased device weight and bulkiness, potentially compromising user comfort and acceptance. The technology is still in its early stages of development, posing challenges in terms of scalability, manufacturing cost, and overall robustness.

## Data Availability

The data that support the findings of this study are available from the corresponding author upon reasonable request.

## Supplementary Materials

Supplementary 1 Movies S1 to S3 Code of execution
